# Smaller farm size and ruminant animals are associated with increased supply of non-provisioning ecosystem services

**DOI:** 10.1007/s13280-022-01726-y

**Published:** 2022-04-16

**Authors:** Johan O. Karlsson, Pernilla Tidåker, Elin Röös

**Affiliations:** Department of Energy and Technology, Swedish University of Agricultural Sciences, Box 7032, 75007 Uppsala, Sweden

**Keywords:** Agriculture, Ecosystem service, Farm, Indicator, Landscape, Livestock

## Abstract

To balance trade-offs between livestock’s negative environmental impacts and their positive contributions (e.g. maintaining semi-natural grasslands, varied agricultural landscapes and crop rotations), a better understanding is needed of how the supply of ecosystem services differs across farms. We analysed a suite of indicators for non-provisioning ecosystem services on a large subset of Swedish farms (71% of farms, covering 82% of agricultural land) and related these to farm type, farm size and livestock density. The analysed indicators exhibited clear geographical patterns with hotspots especially in less productive regions. Controlling for this spatial variation we still found that small-scale and ruminant farms were associated with more varied landscapes, small-scale habitats, semi-natural grasslands and better crop sequences compared to nearby farms specialised in crop production, while farms specialising in monogastric livestock were associated with less varied landscapes and inferior crop sequences. Results for cultural ecosystem services indicated that farms with more semi-natural grassland were associated with more visitors and more likely located within designated recreation or nature conservation areas.

**Supplementary Information:**

The online version contains supplementary material available at 10.1007/s13280-022-01726-y.

## Introduction

Ecosystem services (ES) are the manifold benefits humans obtain from well-functioning ecosystems (Millennium Ecosystem Assessment 2005; IPBES 2019). At landscape scale, ES arise in interlinked social–ecological systems where natural preconditions, human influences (land use and management) and availability of human assets, institutions and demand all contribute to the final realisation^1^ of ES to the benefit of humanity (Plieninger et al. 2015; IPBES 2019 chap. 2.3). Agricultural landscapes, shaped by climate, topography, hydrology and soils, together with agricultural practices and other human influences, therefore exhibit distinct configurations of ES (Raudsepp-Hearne et al. 2010; Qiu & Turner 2013; Andersson et al. 2015; Queiroz et al. 2015).

In Sweden and many other parts of Europe, livestock farming, especially ruminant farming, has strongly influenced the agricultural landscape over time, giving rise to semi-natural grasslands and other semi-natural landscape elements with high levels of biodiversity (Eriksson & Cousins 2014). Such landscapes provide multiple ES, including habitats for pollinating insects (Öckinger & Smith 2007) and natural enemies of pests (Bianchi et al. 2006; Alignier et al. 2014), as well as non-material services supporting cultural identity and recreation (Lindborg et al. 2008; Marzetti et al. 2011; Bengtsson et al. 2019). With increasing agricultural mechanisation in the late twentieth century, agriculture in most European countries, including Sweden, transitioned towards fewer, larger farms, increased specialisation and geographical concentration (Josefsson 2015; de Roest et al. 2018). In the agriculture-dominated plains region of Sweden arable farming has intensified, while farming in less productive forest-dominated regions has come to be dominated by ruminant livestock and perennial grass/legume ley cultivation. In the latter regions, farmland abandonment has been and is still common (Wallander et al. 2019). This specialisation in either crops or livestock is in stark contrast to the predominantly mixed farms of the early twentieth century (de Roest et al. 2018). For example, in the late 1920s, 98% of all Swedish farms had dairy cows (Martiin 2017), compared with only 5% in 2020 (Swedish Board of Agriculture 2020). In parallel, the area of semi-natural grassland declined from 1.3 million ha (Kumm 2003) to less than 0.5 million ha (Swedish Board of Agriculture 2021). These changes have led to landscape homogenisation and simplification, with negative implications for farmland biodiversity and the supply of related ES (Wallander et al. 2019; Clough et al. 2020).

There is now growing consensus in the scientific community that high animal-source food consumption in the Global North needs to decrease if the environmental impacts of food systems are to stay within planetary boundaries (Willett et al. 2019; Clark et al. 2020). Meat and milk from ruminant livestock is particularly resource-intensive and associated with major environmental impacts (Poore and Nemecek 2018). Thus, the number of ruminant livestock needs to decline and livestock farmers need to produce more crops for direct human consumption, in order to reduce environmental impacts and make more macro- and micronutrients available for human consumption per unit land (Foley et al. 2011; Karlsson and Röös 2019). However, this risks exacerbating losses of non-provisioning ES from agricultural landscapes (Moberg et al. 2021).

Without grazing animals, many ES currently provided by semi-natural grasslands and other landscape elements that rely on grazing livestock may be lost (Ford et al. 2012; Bengtsson et al. 2019; Johansen et al. 2019). Moreover, reduced inclusion of perennial forage crops in crop sequences may diminish the supply of ES related to soil formation, nutrient retention and prevention of crop diseases, pests and weeds (Albizua et al. 2015; Martin et al. 2020). Maintaining agricultural activities in less productive regions, often dominated by ruminant livestock farms, is another important priority to preserve biodiversity and cultural values dependent on semi-natural grasslands and agriculture–forest mosaics (Berg 2002; Eriksson et al. 2002), which are more common in these regions. To reconcile necessary cutbacks in livestock farming with maintenance and development of diverse agricultural landscapes and related non-provisioning ES, it is crucial to understand how the supply of non-provisioning ES varies across different types of livestock farms.

As ES are difficult to measure directly, ES assessments generally rely on indicators or proxies derived from empirical data (Haines-Young et al. 2012; Schröter et al. 2021). For assessments with large spatial coverage, reliance on secondary data is often the most feasible option and thereby assumptions on e.g. the validity of indicators and the suitability of secondary data are required (Schröter et al. 2021). Nonetheless, such approaches have previously provided valuable insights into synergies and trade-offs in ES from agricultural landscapes (Raudsepp-Hearne et al. 2010; Turner et al. 2014; Andersson et al. 2015; Queiroz et al. 2015). However, few studies have explicitly linked characteristics of individual farms to outcomes on different ES indicators (a recent exception being Boke Olén et al. 2021), despite farmers being key actors in shaping agricultural landscapes through short- and long-term decisions on farm management.

Against this background, we quantified a suite of indicators of regulating and cultural ES relevant for Swedish agricultural landscapes on a large subset of Swedish farms (71% of farms, covering 82% of agricultural land) and related the results to different farm types, farm sizes and livestock densities. The aim was to determine (1) how non-provisioning ES differ between crop production farms and farms specialising in different livestock enterprises, (2) how farm size and livestock density influence non-provisioning ES and (3) how differences between farms vary geographically. Potential mechanisms behind observed differences were also considered.

## Materials and methods

### Study farms

Study farms (Table 1) were selected from the 2016 Swedish Farm Register (Swedish Board of Agriculture 2018), which includes all farms meeting certain cut-off criteria (*n* = 62 937; criteria include e.g. farming > 5 ha agricultural land or > 2 ha cropland or keeping certain numbers of livestock). Additional criteria applied in this study excluded (i) farms with no cropland (*n* = 2884; which excluded many specialist pig and poultry farms), (ii) farms not matched in the Swedish Integrated Administration and Control System (IACS) database (*n* = 3183; mainly small-scale farms not applying for agricultural support), (iii) farms with pasture, but not reporting any ruminants or horses (*n* = 8474), and (iv) farms for which one or more ES indicators could not be calculated (*n* = 3928; exclusively farms where we could not derive complete crop sequences for at least 50% of cropland area).

**Table 1:** Farms of different types in the 2016 Swedish farm register and included in this study. For each farm type, total agricultural land use and average (avg.) land use per farm is shown, with the area retained on farms selected for this study shown in brackets. For farms included in this study, data on cropland and semi-natural grasslands are also given. Ley refers to grass or grass/legume mixtures grown on cropland, often but not always, in rotation with other crops and harvested by mowing or grazing

Farm type		Swedish Farm Register (2016)			Farms included in this study									
General	Detailed	Number of farms	Agricultural area		Number of farms	Agricultural area		Cropland				Semi-natural grassland		
			Total (kha)	Avg. per farm (ha)		Total (kha)	Per farm (ha)	Total (kha)	Avg. per farm (ha)	Avg. field size (ha)	Ley (% of cropland)	Total (kha)	Avg. per farm (ha)	% of agri. area
Crops	Crop production	17 677	1204	68	12 245 (69%)	810 (67%)	66	789	64	3.7	22	22	1.8	2.7
Ruminants	Dairy cattle	3293	479	145	3248 (99%)	476 (99%)	147	398	123	2.4	69	78	24	16
	Meat cattle	8485	459	54	7872 (93%)	450 (98%)	57	316	40	1.6	77	135	17	30
	Mixed cattle	396	66	168	389 (98%)	66 (99%)	170	54	139	2.9	50	12	30	18
	Sheep	3501	65	19	2669 (76%)	59 (90%)	22	36	14	1.4	85	23	8.6	39
Monogastrics	Pigs	450	58	128	257 (57%)	40 (69%)	155	39	152	5.2	7	0.7	2.6	1.7
	Poultry	180	12	68	51 (28%)	7.1 (58%)	139	6.9	136	5.3	11	0.2	3.6	2.6
Mixed	Mixed livestock	1512	92	61	1353 (89%)	89 (97%)	66	64	48	2.1	58	24	18	28
	Mixed farming	4152	388	93	3860 (93%)	357 (92%)	92	308	80	3.1	35	49	13	14
Small-scale	Small-scale farms	23 291	208	8.9	12 524 (54%)	109 (53%)	8.7	98	7.8	1.4	64	12	0.9	11
All farms	All farms	62 937	3032	48	44 468 (71%)	2464 (81%)	55	2109	47	2.5	45	355	8.0	14

In the Swedish Farm Register, farms are classified based on number of standardised working hours that different farm activities are expected to require (producing different crops, dairy, pig production etc.). Farms are classified as a specific type if more than two-thirds of working hours are spent on that activity. An exception is ‘small-scale farms’, which comprises all farms with less than 400 standardised working hours per year (Swedish Board of Agriculture 2002). ‘Mixed livestock farms’ have no single livestock category accounting for more than two-thirds of working hours, but all livestock do so, while for ‘mixed farms’ neither livestock nor crop production enterprises account for more than two-thirds of working hours.

Each farm was spatially defined by linking farms in the Swedish Farm Register to the IACS database, which contains georeferenced polygons of all agricultural parcels for which farmers have applied for agricultural support and information on e.g. crops sown. Therefore, the term ‘farm’ here refers to agricultural land managed by a certain farm enterprise/farmer (including leased land). For each farm, a study area was defined by applying a 50 m buffer around all agricultural land use parcels tied to that farm. Each farm’s study area is thereby represented by one or several polygons including all agricultural land managed by the farm as well as adjacent land uses and roads necessary for evaluating many of the ES indicators considered. Since the study area was based on each farm’s agricultural land, non-agricultural areas of the farm outside the 50 m buffer (e.g. forests) were not included in the assessment.

### Ecosystem service indicators

**Fig. 1:**
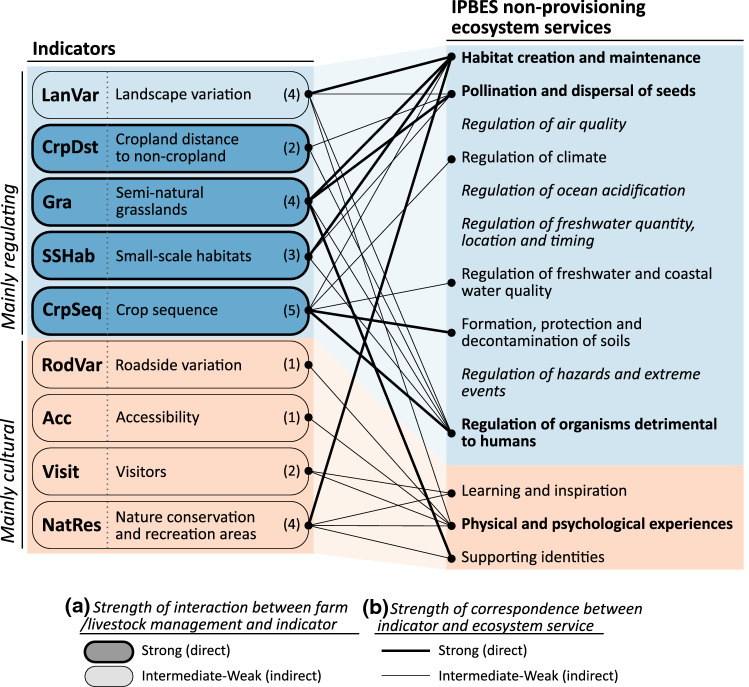
Conceptual framework connecting farm management, indicators and ecosystem services. For each indicator, we qualitatively assessed **a** the strength of interaction between farm/livestock management decisions at farm level and response on the indicator value (indicated by shade and border thickness; a strong interaction means that historic and/or present-day decisions taken on farms have a direct effect on observed indicator values), and **b** ecosystem service(s) to which each indicator relates and how well indicator values can be assumed to correspond to supply of different ecosystem services (indicated by line connections and thickness; a strong correspondence means good scientific evidence for a correlation and that data sources and methods used to calculate the indicator are appropriate). Numbers in brackets indicate the number of ecosystem services connected to that indicator. This figure is intended as an indicative guide to interpret the various indicators in relation to different ES and not as a factual statement on actual correlations

Drawing on previous quantitative indicator frameworks developed to assess trade-offs and synergies between multiple ES from a landscape perspective (e.g. Raudsepp-Hearne et al. 2010; Turner et al. 2014; Andersson et al. 2015; Queiroz et al. 2015), we included nine ES indicators in our assessment (Table 2). Indicators were selected based on relevance for Swedish agricultural landscapes, possibility to calculate from available secondary data with national coverage and potential for automated calculation for large numbers of farms. We also aimed at indicators directly or indirectly influenced by past and/or present farm management. Five of the selected indicators mainly relate to regulating ES and four to cultural ES, and together they cover nine of the 13 non-provisioning ES defined by IPBES (2019) (Fig. 1). Apart from the IACS database, a number of additional datasets (e.g. detailed road and land cover maps and georeferenced photo uploads) were used in quantifying the various indicators (illustrated in Fig. S5). Descriptions of all datasets used together with a detailed description of each indicator and references to literature on their value as proxies for different ES are provided in Supplementary Materials.

**Table 2:** Ecosystem service indicators included in this study. For each indicator a brief description is provided followed by references to studies where similar indicators have been developed and/or used

Indicator		Description
LanVar	Landscape variation	Total length of borders between land cover patches divided by total study area (Andersson et al. 2015)
CrpDst	Cropland distance to non-cropland	Average distance from field interior to nearest non-cropland habitat (excluding water or densely built-up areas) (Andersson et al. 2015; Queiroz et al. 2015)
Gra	Semi-natural grasslands	Area of semi-natural grasslands according to the IACS database with areas also present in the Swedish meadow and pasture inventory, weighed with a factor of two (Andersson et al. 2015)
SSHab	Small-scale habitats	Number of small-scale habitats (e.g. field islets, clearance cairns) within cropland divided by total area of cropland
CrpSeq	Crop sequence	Crop sequence indicator presented in Leteinturier et al. (2006) that takes the order of crops in a sequence, minimum recommended return times and crop diversity into account and calculates a final score. Calculated for a seven-year sequence (2013–2019). In general, diverse sequences with a high proportion of ley generates a high score on this indicator
RodVar	Roadside variation	Number of land cover patches intercepted by/adjacent to roads divided by study area (Andersson et al. 2015)
Acc	Accessibility	Share of study area within 100 m from roads plus population density within a buffer of 10 km around the study area. Both terms scaled prior to summation (Andersson et al. 2015)
Visit	Visitors	Number of unique users uploading photos to *Flicker* plus number of users reporting species observations to *Artportalen (Swedish Species Observation System)* divided by study area. Both terms scaled prior to summation (Raudsepp-Hearne et al. 2010; Turner et al. 2014; Queiroz et al. 2015; Le Clec'h et al. 2019)
NatRes	Nature conservation and recreation areas	Area of nature reserves plus Natura 2000 areas plus areas of national interest for nature conservation and recreation, divided by total study area (Turner et al. 2014; Queiroz et al. 2015)

For each of the nine indicators, we qualitatively assessed the strength of interaction between farm/livestock management and indicator values, the ES to which the indicator relates and how well indicator values can be assumed to capture the supply of ES (Fig. 1). This assessment was performed through a review of relevant literature (references provided in Supplementary Materials) and discussions with experts in the field of biodiversity and ecosystem services. It is intended as an indicative guide to interpret the various indicators in relation to different ES and not as a factual statement on actual correlations. Most indicators are based on biophysical ecosystem characteristics (e.g. land cover) and relate primarily to ES supply, while some aspects of demand are also covered. For example the indicator for visitors to the farms (Visit) can be argued to cover both supply and demand, while for the indicators relating to pollination and biological pest control (LanVar, CrpDst, Gra, SSHab) the farm’s cropland itself constitutes some level of demand, although this will vary with the crops grown and agronomic practices employed (Zulian et al. 2013), aspects not accounted for in the indicators.

### Data processing and indicator exclusion

All calculated indicators were scaled and normalised to zero mean and one standard deviation prior to most analyses. Correlations between different indicators were analysed with Spearman’s rank correlation. Strongly correlated indicators were excluded from the analysis to avoid biassed interpretation of results. We did not set any strict cut-off for this, but rather excluded correlated indicators if there was reason to believe that they reflected similar landscape properties and thus represented redundant information (i.e. due to underlying data and how they were calculated). The indicator to retain was selected based on robustness of calculation, number of ES it was assumed to correspond to (Fig. 1) and previous use in the literature.

All data processing and statistical analysis were performed with *R* (version 4.0.5; R Core Team 2021), primarily using the packages *sf* (Pebesma 2018) for spatial data processing, *dplyr* (Wickham et al. 2021) for general data processing, *correlation* (Makowski et al. 2020) for Spearman’s rank correlation, *rstatix* (Kassambara 2021) for t-tests and effect size estimation, *stats* (R Core Team 2021) for k-means clustering and *mgcv* (Wood 2017) for fitting a generalised additive model (GAM). Custom *R* scripts used to calculate each indicator and process results are available at https://doi.org/10.5281/zenodo.5336914. Data used to prepare Figs. 3, 5, S1 and S2 can be found in Supplementary Materials.

### Defining farm clusters based on farm type, livestock density and size

To analyse the correspondence between different farm characteristics and the assessed indicators, we clustered the farms in three different ways based on either type, livestock density or size. For each set of clusters one was selected as a reference to which the other farm clusters were compared.

Effects of farm type were assessed by clustering farms according to the types used in the Swedish Farm Register (Table 1). Crop production farms were used as reference farms to which other farm types were compared.

Effects of livestock density (i.e. livestock units (LSU) per hectare total agricultural land; Eurostat 2014) were assessed by selecting farms with only one livestock species (cattle, sheep, horses or pigs + poultry) and using a k-means clustering algorithm (Hartigan and Wong 1979) to cluster farms into livestock density groups represented by the group mean. K-means clustering aims to minimise the deviation of each farm’s livestock density from the mean of the cluster to which it belongs. Number of clusters was selected for each livestock species such that the clustering resulted in four clusters with more than 20 farms in each. Clusters with ≤ 20 farms were excluded from the analysis, which resulted in 2, 1, 0 and 35 farms with cattle, sheep, horses and pigs + poultry being dropped, respectively. These were farms with very high livestock densities. Farms keeping a mix of livestock species were also excluded from this analysis. The different livestock density clusters were compared with reference farms with zero livestock units. This way of grouping the farms was in order to study the effects of density of different livestock species on indicator values without confounding effects of farms keeping a mix of livestock species.

Effects of farm size were analysed following a similar procedure, with all farms clustered into five size clusters using the k-means clustering algorithm. The most populous cluster (which comprised the smallest farm sizes) was used as reference farms to which the other four farm size clusters were compared.

### Comparing farms to surrounding reference farms

Many of the indicators were expected to exhibit spatial autocorrelation, due to e.g. varying morphology, climate, soils and demography. Different regions of Sweden also differ in terms of which types of farms are more common, with e.g. specialised crop production farms being more common in the agriculturally dominated plains regions and farms specialising in ruminant production being more common in the forest-dominated regions. To control for these spatial effects, we analysed effects of farm type, livestock density and farm size on a farm’s indicator values in relation to surrounding farms, where regional conditions can be assumed more similar. This was done by setting a 20-km radius around each farm’s centre point and calculating the difference ($D$) in its scaled indicator values from the area-weighted mean values of reference farms within this radius according to Eq. (1). Here $I$ is the farms scaled indicator value and $i$ and $a$ is the scaled indicator value and total agricultural area, respectively, for each reference farm within the 20-km radius.

1 $$D = I - \frac{\sum i \times a}{{\sum a}}$$

As there is a correspondence between farm size and several of the indicators, the area weighting causes mean *D* to deviate from zero also for the reference farms cluster. Welch’s unequal variances *t* tests were used to test for statistical significance (α = 0.01) of observed differences in mean *D* for the different farms clusters compared with the reference farms cluster. Effect size was estimated using Cohen’s *d* (*d*), with a variance term mirroring that in Welch’s *t* test (Aoki 2020). Effect size was categorised as large ($\left| d \right| > 0.8$), moderate ($\left| d \right| > 0.5$), small ($\left| d \right| > 0.2$) or otherwise negligible (Cohen 2013).

### Generalised additive modelling

For the landscape variation indicator (LanVar), we also fitted a generalised additive model (GAM). The final model included livestock density for cattle, sheep, horses and monogastric animals (pigs + poultry), geographical location and logarithm of total farm size as smooth terms. We used thin-plate regression splines for livestock densities and farm size, and Duchon splines for geographical location together with the fast REML smoothing parameter estimation method with discretisation of covariates. A scaled *t*-error distribution model was used with an identity link. Since the data contained some extreme outliers in terms of livestock density, we excluded the 0.05% of farms with the highest densities of each animal (*n* = 91) prior to fitting the model. Residuals were checked for normality and the dataset was split into a training set (75% of data) used to fit the model and a validation set (25% of data) used to evaluate model performance and any prediction bias.

## Results

Our analysis revealed that ruminant, mixed and small-scale farms were associated with more varied landscapes (LanVar), semi-natural grasslands (Gra) and small-scale habitats (SSHab) and better crop sequences (CrpSeq) than crop production farms. Monogastric livestock farms were associated with less varied landscapes, small-scale habitats and lower score on the crop sequence indicator compared to crop production farms. Small-scale farms were more accessible (Acc), while monogastric livestock farms were less accessible, than crop production farms. For the other indicators relating to cultural ES, we found higher indicator values on small-scale farms (Visit, NatRes), ruminant farms (NatRes) and mixed farms (Visit, NatRes) with a statistically significant difference from crop production farms, but effect sizes were negligible except for NatRes on mixed farms.

We found mainly positive correlations between the different indicators, indicating that there are few trade-offs between these indicators (Table 3). There was strong positive correlation between LanVar, CrpDst and RodVar, which is likely an effect of diversity/complexity in land cover affecting these indicators in a similar way. There is thus a risk that these indicators all indicate for similar landscape properties and including all in the assessment might lead to a biassed assessment. To avoid this we chose to retain LanVar, which had the broadest coverage in terms of ES captured, and omit CrpDst and RodVar from any further analysis. There was also a moderate positive correlation between LanVar and SSHab and Acc, but we chose to retain all these indicators since they relate to diverging landscape properties.

**Table 3:** Correlation between indicator pairs. Numbers show Spearman’s Rho (*r*_s_) for statistically significant (*p* < 0.01) correlations. Bold indicates moderate positive correlation (*r*_s_ ≥ 0.3) and underline indicates strong positive correlation (*r*_s_ ≥ 0.5). Indicators excluded from further analysis are in italics

CrpDst	Gra	SSHab	CrpSeq	Acc	RodVar	Visit	NatRes	
***0.86***	0.18	**0.32**	0.12	**0.35**	***0.62***	− 0.02		LanVar
	*0.27*	***0.34***	*0.12*	***0.32***	***0.52***	− *0.05*	− *0.04*	*CrpDst*
		0.11	0.22	0.03	*0.09*	0.19	0.17	Gra
			0.06		*0.27*	− 0.02	− 0.02	SSHab
				0.02	*0.10*	0.03	0.07	CrpSeq
					*0.15*	0.14		Acc
						− *0.05*	− *0.03*	*RodVar*
							0.26	Visit

**Fig. 2:**
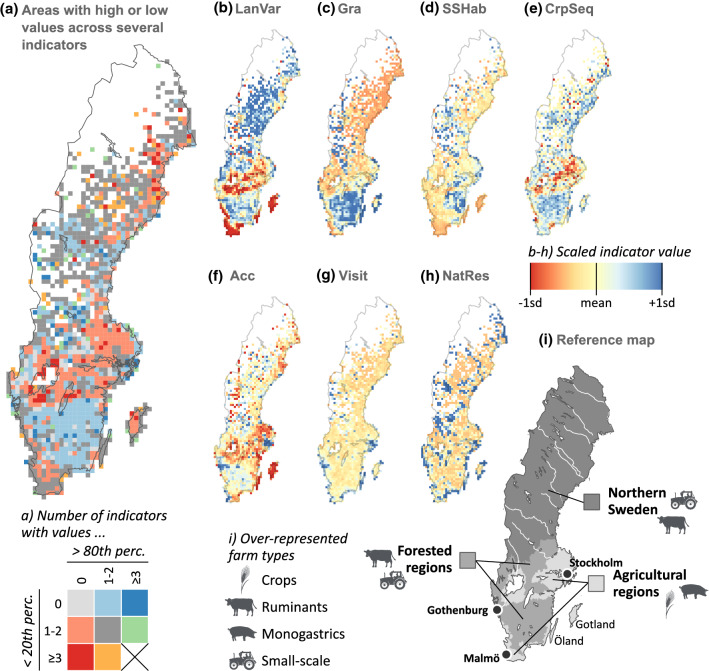
Maps showing regional patterns in indicator values on a 15 km × 15 km grid. **a** Number of indicators within each grid cell with a value above the 80th percentile and below the 20th percentile. **b**–**h** Scaled indicator values for each indicator. The value of each grid cell represents the area-weighted mean value for all farms within that grid cell. **i** Reference map with the three regions used in assessments. For each region, more common farm types compared with Sweden as a whole are indicated

The indicators showed clear geographical patterns. Landscape variation (Fig. 2b) was generally higher in forested regions and northern Sweden and lower in agricultural regions and especially in southernmost parts of Sweden. The indicator for small-scale habitats (Fig. 2d) displayed similar patterns but with more pronounced hotspots, especially in south-eastern forested regions, along the east coast north of Stockholm and in north-western Sweden. The indicator for semi-natural grasslands (Fig. 2c) showed high values in southern forested regions, the island of Öland and alpine regions of north-western Sweden. The crop sequence indicator (Fig. 2e) showed low values in agricultural regions in central Sweden, while forested regions and the east coast of northern Sweden showed high values due to more frequent inclusion of ley in crop sequences. As expected, accessibility (Fig. 2f) exhibited high values in more densely populated regions, but also generally lower values in agricultural regions due to larger fields and thus a lower proportion of agricultural area within 100 m of roads. The results for visitors (Fig. 2g) and nature reserves (Fig. 2h) were more scattered, with hotspots close to coasts, waterbodies, the major cities of Stockholm, Gothenburg and Malmö, and the islands of Öland and Gotland.

The aggregated results (Fig. 2a) showed that forested regions in southern Sweden performed best for the indicators assessed, with large areas with at least one indicator above the 80th percentile and almost no areas with indicators below the 20th percentile. In contrast, agricultural regions in central Sweden had large areas with one or more indicators below the 20th percentile and no indicators above the 80th percentile.

### Landscape variation (LanVar)

**Fig. 3:**
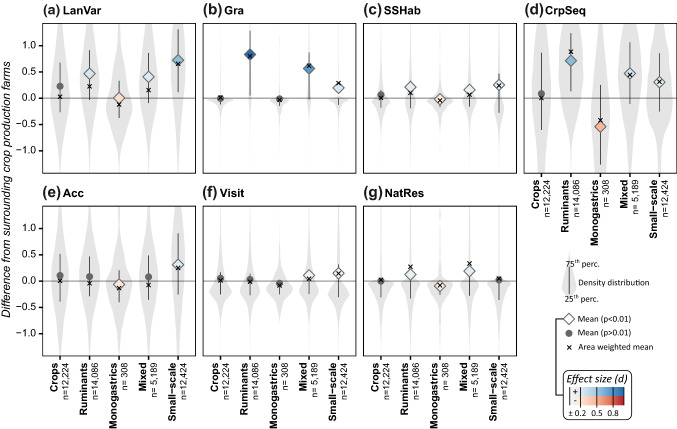
Effect of farm type on indicator values. The y-axis show the difference in indicator value compared with the area-weighted mean value for specialist crop production farms (‘Crops’) within a 20-km radius. For each farm type, the interquartile range (error bars), mean (diamonds/circles) and area-weighted mean (crosses) are shown. Diamonds indicate a statistically significant difference in mean difference from the ‘Crops’ group (Welch’s *t* test; *p* < 0.01) and colour indicates effect size (Cohen’s *d*). Note: Mean difference also deviated from zero for the reference farms, due to the area weighting and stochastic effects from how farms are distributed in the landscape

**Fig. 4:**
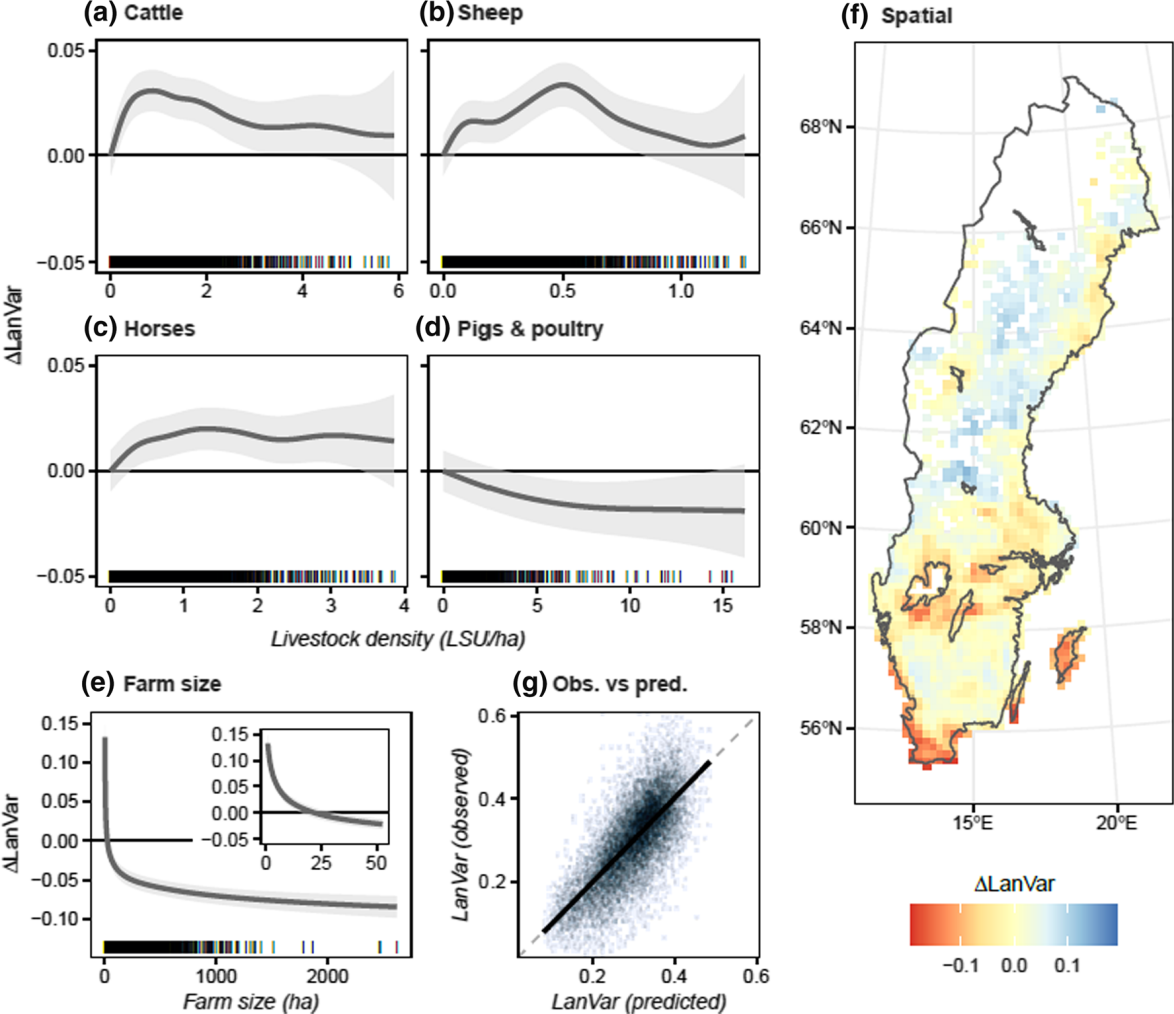
Results from the GAM with LanVar as dependent variable and livestock density, farm size and geographical location as independent variables. Panels (**a**–**f**) show model response to changes in livestock density (**a**–**d**), farm size (**e**) and geographical location (**f**) with the other covariates held constant. Model responses are expressed as difference in LanVar from a 22 ha farm (i.e. the median farm size) with zero livestock units located in the Stockholm area. The shaded areas indicate 95% confidence bounds and bars at the bottom of each panel show individual data points (farms) in the training dataset. Panel (**g**) shows observed vs predicted values when predicting on new data. The dashed grey line represents the one-to-one line and the solid black line is the linear regression between predicted and observed values

Compared with surrounding crop production farms, we found higher values of LanVar for ruminant (Cohen’s *d* = 0.32), mixed (*d* = 0.25) and small-scale farms (*d* = 0.60) and lower values for monogastric farms (*d* = − 0.34; Fig. 3a). On disaggregating the ruminant farm category, the largest difference was found for sheep farms (*d* = 0.61) while no statistically significant difference was found for dairy and mixed cattle farms (Fig. S1a). The GAM revealed a clear negative response on LanVar with increasing farm size, especially in the range 0–50 ha (Fig. 4e). Controlling for farm size and geographical location revealed a positive relationship between density of ruminants and horses and LanVar (Fig. 4a–c). The response was largest for relatively low livestock densities and levelled off or decreased as livestock density increased. Monogastric livestock density gave a negative response in the model (Fig. 4d). The response magnitudes indicated that farm size and geographical location were stronger explanatory variables than livestock density in the model. The model explained 50% of deviance in LanVar and using new data as input resulted in unbiassed predictions (Fig. 4g), including when predicting for different farm types and geographical locations (data not shown).

### Semi-natural grasslands (Gra)

**Fig. 5:**
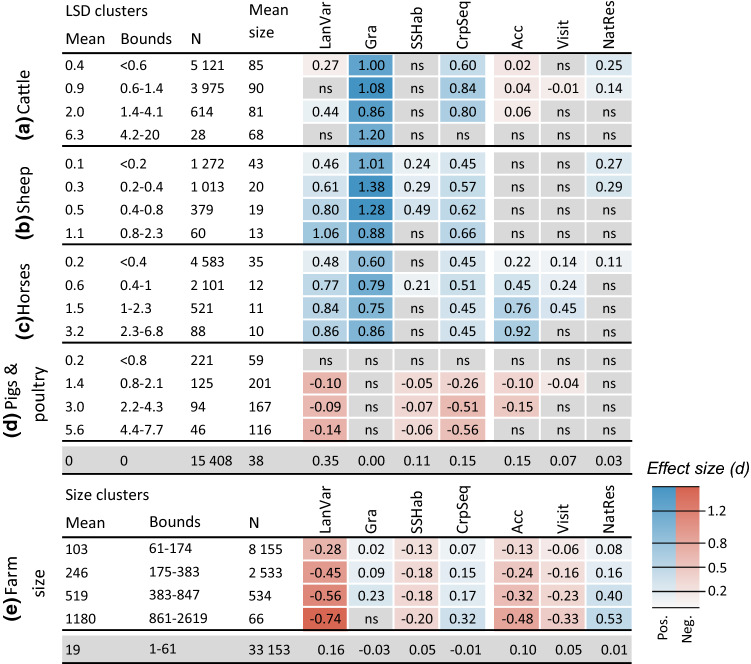
Effects of livestock density and farm size on indicator values. Values shown are mean difference in indicator value from the area-weighted mean of surrounding reference farms within a 20-km radius. Numbers are only presented for cases with a statistically significant difference in mean difference from the reference farms (Welch’s *t* test; *p* < 0.01) and colour indicates effect size (Cohen’s *d*). For livestock density (**a**–**d**) farms within each cluster are compared to farms without livestock (reference farms). Each cluster consists of farms keeping only a single livestock category (cattle, sheep, horses or pigs + poultry) and each cluster’s mean livestock density (LSD; LSU/ha), minimum and maximum LSD of farms in the cluster (Bounds), number of farms (*N*) and mean farm size (ha) are presented. For farm size (**e**) farms within each cluster are compared to farms in the smallest farm size cluster (reference farms). For each cluster mean farm size (ha), minimum and maximum farm size in the cluster (Bounds) and number of farms (*N*) is presented. Note: Mean difference deviates from zero also for reference farms, due to the area weighting and stochastic effects from how farms are distributed in the landscape

As expected, ruminant, mixed and small-scale farms showed higher values of Gra than surrounding crop production farms (*d* = 1.1, 0.82 and 0.31, respectively; Fig. 3b), with the largest effect size observed for sheep farms (*d* = 1.3; Fig. S1b). Analysis of different livestock density clusters revealed that the largest difference from surrounding farms without livestock occurred for clusters with mean livestock density around one LSU/ha for cattle and 0.5 LSU/ha for sheep and horses, beyond which the difference levelled off or decreased (Fig. 5a–c).

### Small-scale habitats (SSHab)

Compared with surrounding crop production farms, ruminant, mixed and small-scale farms had higher values of SSHab, although effect size was negligible except for small-scale farms (*d* = 0.18, 0.15 and 0.21, respectively; Fig. 3c). The largest effect sizes were found for mixed livestock and sheep farms (*d* = 0.24 and 0.27, respectively; Fig. S1c), while no statistically significant differences were found for dairy and mixed cattle farms. We also observed lower SSHab values for the larger farm size clusters compared with surrounding farms in the smallest size cluster (*d* = − 0.23–0.33; Fig. 5e).

### Crop sequence (CrpSeq)

The value of CrpSeq was higher on ruminant, mixed and small-scale farms compared with surrounding crop production farms (*d* = 0.62, 0.36 and 0.20, respectively; Fig. 3d), while monogastric farms showed lower values (*d* = − 0.53). The largest difference was observed for dairy cattle farms (*d* = 0.80; Fig. S1d). Comparing the different livestock density clusters to surrounding farms without livestock, we found that the differences tended to level off or decrease above approximately one LSU/ha for cattle and 0.5 LSU/ha for sheep and horses (Fig. 5a–c). We also observed higher values for the larger farm size clusters compared with surrounding farms in the smallest size cluster, but effect size was negligible except for the largest farm size cluster (moderate effect size, *d* = 0.09–0.36; Fig. 5e).

### Accessibility (Acc)

Compared with surrounding crop production farms, small-scale farms had higher values of Acc and monogastric farms had lower values (*d* = 0.23 and − 0.23, respectively; Fig. 3d), while no statistically significant difference was found for other farm types. The largest difference was found for farms with only horses compared with farms without livestock (*d* = 0.07–0.64; Fig. 5c). Larger farms tended to have lower values of Acc than surrounding farms in the smallest size cluster, and absolute effect size increased with mean farm size of the cluster from small to moderate (*d* = − 0.30–0.81; Fig. 5e).

### Visitors (Visit)

**Fig. 6:**
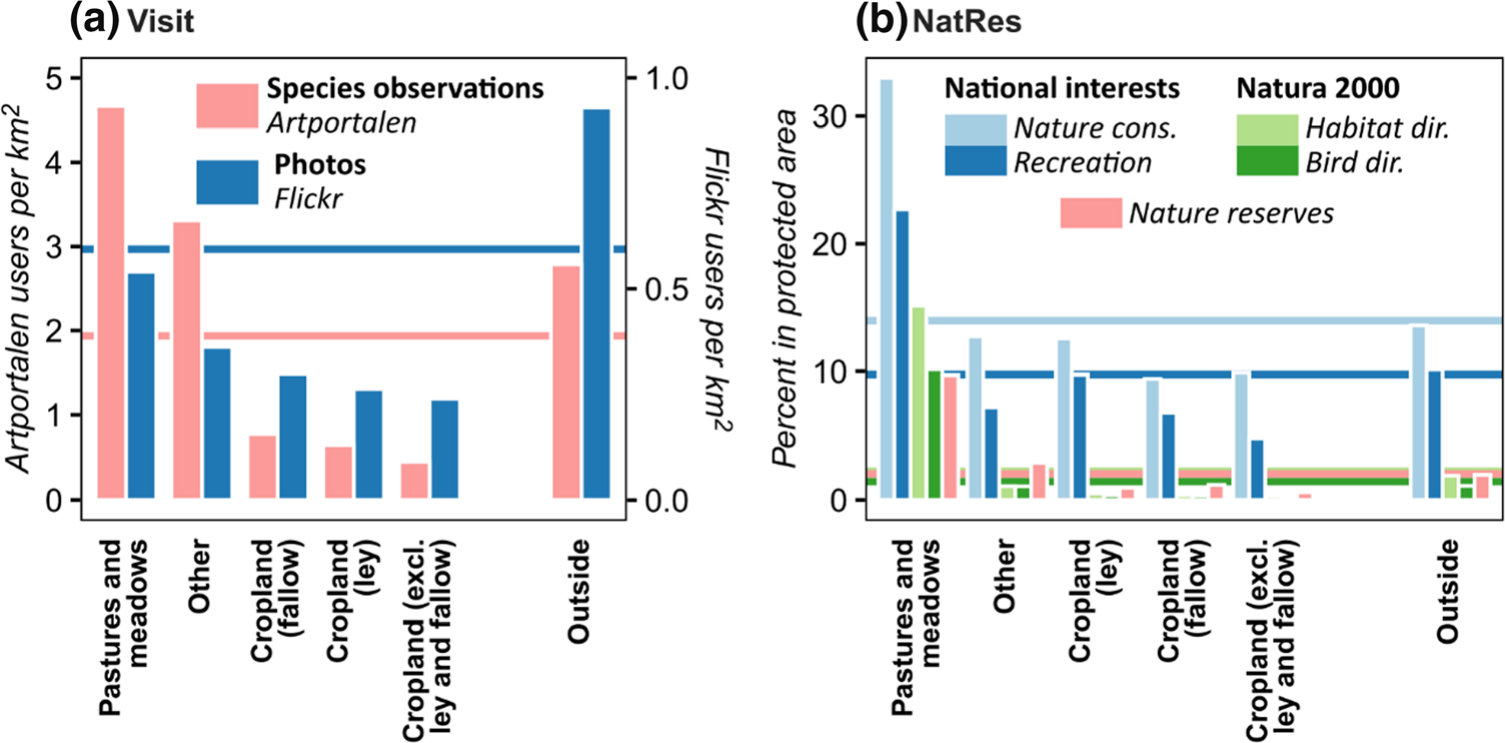
**a** Number of users reporting species observations (left-hand bars) and uploading photos (right-hand bars) per km^2^ for different agricultural land uses. **b** Percentage of different agricultural land uses falling within (from left to right) areas of national interest for nature conservation and recreation, Natura 2000 areas under the EU Habitats and Birds Directives, and nature reserves. Agricultural land use was evaluated for 2016. The category ‘Other’ refers to e.g. uncultivated field margins and wetlands on agricultural land. ‘Outside’ refers to land within a farm’s study area that is not part of the farm’s agricultural land. The horizontal lines show the average for all land uses within farm study areas (including non-agricultural land)

Mixed and small-scale farms showed higher and monogastric farms lower values of Visit than surrounding crop production farms, but effect sizes were negligible (*d* = 0.06, 0.09 and − 0.14, respectively; Fig. 3f). The largest effect sizes were observed for farms with horses compared with surrounding farms without livestock (*d* = 0.07–0.29; Fig. 5c). A correlation was found between Gra and Visit (Table 3), indicating that farms with semi-natural grasslands attract more visitors. It is, however, not certain that the semi-natural grasslands per se attract more visitors. Calculation of total number of visitor points per square kilometre for different land uses (Fig. 6a) did, however, strengthen that hypothesis by revealing that semi-natural grasslands received more visitors than all other agricultural land uses. This was especially the case for species observations, where visitor counts for semi-natural grasslands were more than twice the average for all land uses.

### Nature conservation and recreation areas (NatRes)

Mixed and ruminant farms showed higher NatRes values, and monogastric farms lower values, compared with surrounding crop production farms (*d* = 0.21, 0.14 and − 0.15, respectively; Fig. 3g), with sheep and mixed livestock farms having the largest effect sizes (*d* = 0.22 and 0.27, respectively; Fig. S1g). Larger farm sizes corresponded with higher values of NatRes compared to surrounding farms in the smallest size cluster, with effect size increasing with farm size (*d* = 0.08–0.46; Fig. 5e). NatRes was also positively correlated with Gra (Table 3) and the share of semi-natural grasslands within different protected areas was much higher than for all other land uses within farm study areas (Fig. 6b).

## Discussion

### Small-scale farms and farms with ruminants and horses are associated with more varied agricultural landscapes

Smaller farms tended to be associated with higher landscape variation and more small-scale habitats, as found previously by Levin (2006) in Denmark (smaller fields and more small-scale habitats on farms < 25 ha). Smaller mean field size correlated strongly with our indicator for landscape variation (*r*^2^ = 0.46; Fig. S3b). Belfrage et al. (2015) also found a strong negative correlation between farm size and on-farm landscape heterogeneity (measured with Shannon–Wiener diversity index) for farms in southern Sweden with similar surrounding landscape heterogeneity. They also noted that open ditches, field islets and stone cairns were common on smaller farms, while on larger farms most ditches were covered and obstacles had been removed. Levin (2006) suggested that the difference between farm sizes may be explained by differing motivations among farmers managing small or large farms. Smaller farms are commonly operated by part-time or hobby farmers, who have other major sources of income, while larger farms tend to be operated by full-time farmers, for whom cost minimisation by rationalising fields (i.e. merging fields and removing obstacles) is more important. Over time, these differences in incentives would result in the situation observed today, with smaller fields and more diverse landscapes associated with small farms. We also noted that the negative response of increased farm size levelled off above around 50 ha (Fig. 4e), beyond which the proportion of full-time farmers is high, strengthening this hypothesis. An alternative hypothesis is that small farms are concentrated to less attractive tracts of land where field rationalisation is difficult. Likely, both of these hypothesis have merit and their respective explanatory power may differ regionally.

These results confirm previous findings, highlighting the importance of supporting small-scale farmers when seeking to maintain diverse agricultural landscapes and related ES. This is not being achieved currently, e.g. between 2007 and 2016 the majority of farmland abandonment in Sweden occurred on farms operated by non-full-time farmers in less productive regions (Swedish Board of Agriculture 2017a). To turn this development, support payments could be redirected towards the less productive regions (Brady et al. 2017) or targeted payments for small fields could be implemented through the national strategic plans within the new Common Agricultural Policy (Clough et al. 2020).

Farms with ruminants and horses were positively associated with higher landscape variation and, to a lesser extent, small-scale habitats in cropland. A potential explanation is that a large proportion of cropland area is devoted to ley cultivation on these farms. An important incentive when rationalising fields is to minimise the time spent on machine operations per hectare, thereby reducing costs (Clough et al. 2020). Fewer field operations for ley compared with arable crops make ley cultivation less sensitive to structurally suboptimal fields, thereby reducing economic incentives to invest in field rationalisation (Wästfelt and Eriksson 2017). This has been observed previously e.g. for central Sweden, where increases in field size between 1990 and 2014 were more pronounced on farms specialising in cereal production compared with ruminant livestock farms (Wästfelt and Eriksson 2017). We found no statistically significant differences in LanVar and SSHab between dairy or mixed cattle farms and crop production farms (Fig. S1a, c), even though these farms cultivate ley on a large proportion of their cropland. This is likely because these farms are larger and have more diverse crop sequences (Fig. S4a) than other categories of ruminant farms, and thus likely use larger machinery, making large, rational fields more important.

Unsurprisingly, ruminant farms were strongly associated with semi-natural grasslands, but increased livestock density beyond a certain threshold did not seem to be associated with a higher score on the indicator for semi-natural grasslands (Fig. 5a–c). This shows that number of ruminant animals is not the only factor limiting grazing of semi-natural grasslands on many farms, as also concluded by Larsson et al. (2020). It is also in line with scenario studies showing that the current area of semi-natural grassland could be managed with much fewer animals and greenhouse gas emissions if animals were suitably dispersed in the landscape and in semi-natural pasture-based production systems (Röös et al. 2016; Karlsson and Röös 2019). However, to reach conservation goals for Swedish grassland biomes, the area and quality of semi-natural grassland both need to increase (Swedish Environmental Protection Agency 2020). Based on our results, increased ruminant livestock production alone would not be sufficient and economic incentives to increase the share of animals grazing semi-natural grasslands would be needed. This could be achieved through e.g. more effective environmental payment schemes (Larsson et al. 2020), better price premiums (Holmström et al. 2021) or innovative practices that minimise costs (e.g. merging of small scattered pasture into large pasture–forest mosaics; Holmström et al. 2018).

Interestingly, we found a strong positive association between horse farms and our indicator for semi-natural grasslands. Effect size (*d* = 0.86–1.1) was slightly smaller than for cattle (*d* = 1.1–1.6) and sheep (*d* = 1.1–1.6), but indicated that horses still make a significant contribution to grazing Swedish semi-natural grasslands. The number of horses in Sweden is estimated to be around 350 000 (Swedish Board of Agriculture 2017b), of which less than one-third are represented in the Swedish Farm Register. Whether and to what extent the remaining two-thirds contribute to grazing semi-natural grasslands was thus beyond the scope of our assessment. When using horses for grazing, it should be noted that historical management regime is important for the site-specific plant and animal communities in semi-natural grasslands (Bonari et al. 2017). For optimal biodiversity conservation outcomes, management should ideally mimic the historical management regime, which generally comprised haymaking or grazing by cattle and sheep. Nonetheless, grazing horses have been shown to preserve biodiversity values (Köhler et al. 2016; Saastamoinen et al. 2017; Garrido et al. 2019), including pollinator habitats (Garrido et al. 2019) in semi-natural grasslands, and could potentially do so with lower methane emissions compared with ruminants.

### Farms with ruminants or horses and large farms are associated with better crop sequences

Ruminant farms were clearly associated with higher values of our crop sequence indicator, due to a larger proportion of ley in the crop sequences (Figs. 3a, S4a). However, effect size did not increase with increasing livestock density, but rather levelled off at relatively low densities (Fig. 5a–c), and all density clusters except the lowest were similar in terms of crop diversity and inclusion of ley (Fig. S4c). This could indicate higher productivity on more densely stocked farms, but possibly also greater reliance on bought feed (including roughage). We noted fairly high mean inclusion of ley in crop sequences on farms without livestock (47%; Fig. S4c), which may partly be explained by grass and clover seed production, but these farms likely also provide feed for ruminants or horses on other farms. This highlights the interdependence of farms, where e.g. farm animals on one farm can affect crop sequences and related ES on farms without livestock.

Larger farms were associated with higher values of the crop sequence indicator (Fig. 5e). While smaller farms tended to cultivate more ley, this was compensated for by more diverse crop sequences on larger farms (Fig. S4b). This contradicts the perception that crop diversity is higher on small farms, as observed by e.g. Ricciardi et al. (2021) on a global level and Belfrage et al. (2005) in Sweden. However, e.g. Persson et al. (2010) also found a positive association between mean field size (which correlates with farm size) and crop diversity in southern Sweden and Weigel et al. (2018) found more diverse crop portfolios on larger farms in Bavaria (Germany). A potential explanation is that larger farms have better access to machinery that can handle a greater diversity of crops.

### Farms with horses and semi-natural grasslands are associated with indicators for cultural ecosystem services

Results for the indicators relating to cultural ES were less conclusive, with generally small or negligible effect sizes. An exception was that horse farms in higher livestock density clusters showed higher values for accessibility with a moderate effect size (Fig. 5c). These clusters likely represent farms focussing on recreational horseback riding and were often located in areas with high population density (data not shown). This also seemed to be linked with more visitors on horse farms, although effect sizes were small or negligible (Fig. 5c). Proximity of an area to home has previously been shown to be an important factor for outdoor recreation (Fagerholm et al. 2019), with de Vries and de Boer (2008) noting that “farmland areas are more visited because of their nearness than of their high quality”. We observed a correlation between population density around a farm and Visit (*r*_s_ = 0.16; data not shown) but correlation coefficients were stronger between Visit and Gra and NatRes (*r*_s_ = 0.19 and 0.26, respectively; Table 3), suggesting that landscape quality does matter. It should be noted that the data sources used here to quantify visitors to the farms are not fully representative of the Swedish population, e.g. people reporting species observations to *Artportalen* are likely to have a strong interest in nature and wildlife, which might make them willing to travel farther than the average Swede for outdoor recreation. It is also likely that observers are more motivated to report rare species, which would bias the visitor counts towards areas with rare species. The most distinct pattern we observed for visitors was a negative effect for larger farm size clusters (Fig. 5e). Considering that farm size was positively associated with Gra and NatRes, both of which presumably indicate visitor attractiveness, as confirmed in our correlation analysis (Table 3), and that larger farms tended to be located in areas with higher population density (data not shown), this effect is likely related to larger farms being less accessible, with larger fields and more limited road access than smaller farms.

Farms with ruminants tended to score higher on our indicator for nature conservation and recreation areas compared with surrounding crop production farms, indicating that the landscape on and around these farms has higher nature conservation and recreation values. Effect sizes were generally small or negligible, but we observed a large difference between semi-natural grasslands and other agricultural land uses, both in terms of number of visitors (Fig. 6a) and the fraction within protected areas (Fig. 6b), which highlights the value of these areas for nature conservation and recreation. We found larger effect sizes for NatRes in agricultural regions than in other regions (Fig. S2g). This was also reflected in the fraction of semi-natural grasslands protected, with e.g. 15% located in nature reserves in agricultural regions compared with 3–5% in other regions (data not shown). This may indicate that semi-natural grasslands are considered more important to protect in cropland-dominated regions. We did not observe a similar regional difference for the share of cropland in protected areas.

### Perspectives cutting across the suite of ecosystem service indicators

We found the highest values for most indicators in less productive forest-dominated regions of Sweden (Fig. 2), which highlights the value of these agricultural landscapes for non-provisioning ES and the importance of stopping ongoing farmland abandonment there. On the other hand, we generally observed larger positive effect sizes for ruminant, mixed and small-scale farms in agricultural regions compared with forested regions and northern Sweden (Fig. S2). This is likely due to agricultural intensification and rationalisation being more pronounced in the agricultural regions, while in forested regions and northern Sweden the trend has rather been for farmland abandonment and reduced arable crop production, resulting in larger differences between individual farms in the agricultural regions.

Farms with ruminants and horses showed higher values for many of the indicators compared with crop production farms, highlighting the association between grazing animals and diverse agricultural landscapes and related ES. However, we also detected a “saturation effect” for several indicators whereby increased livestock density beyond a certain threshold was not associated with higher indicator values. Moving forward, reduced stocking density on farms with high livestock densities might therefore be a win–win solution, reducing absolute livestock numbers and thereby resource use and environmental impacts, while avoiding loss of agricultural landscape diversity and ES. Reduced stocking densities may also facilitate manure recycling and reduce nitrogen losses. It is important to ensure continued presence of grazing animals in regions currently dominated by crop production, where their positive contribution was largest, while at the same time supporting crop production in livestock-dominated forested regions to avoid farmland abandonment as an effect of fewer ruminants. We observed only negative or no effects on the seven indicators analysed for farms specialising in monogastric livestock production, showing that pig and poultry are animal-source food production enterprises that could be reduced without losing agricultural landscape diversity and related ES. It is also important to note that for most indicators, there was a large spread in observed values for individual farms, independent of farm type, size or livestock density.

Our results showed associations between different farm types and livestock densities and the seven indicators assessed, but these do not necessarily reflect causality. It is also important to bear in mind that causal links may change over time, e.g. the observed positive association between small farms or farms with ruminants and horses and landscape variation and small-scale habitats is a result of landscape change over long periods and under varying regulatory environments. Thus, while there may have been causal links between farms specialising in arable crop production and landscape homogenisation and increased field size, such links may be broken through different policies and regulations. For example, many small-scale habitats in the agricultural landscape (e.g. field islets, clearance cairns, stone walls) are today protected under Swedish environmental law (5 § SFS 1998:1252), which makes it difficult for farmers to remove such features. Many of the observed associations are also due to current economic incentives that may change in the future. For example, perennial ley is today grown mainly on livestock farms but may become more economically feasible on arable farms if e.g. fertiliser prices increase or if other outlets such as bioenergy production become more lucrative (Koppelmäki et al. 2019). Europe has recently experienced surging fertiliser prices due to natural gas shortage (Fedorinova et al. 2022) and a future transition towards renewable synthetic nitrogen could also push up prices (Tallaksen et al. 2015), potentially making nitrogen fixating crops a more viable alternative.

## Conclusions

Our results confirmed previous findings that small-scale farms and farms in less productive regions maintain fine-grained agricultural landscapes and related ES. Supporting these farms should therefore be a key policy priority. We also found associations between farms with ruminant animals and many of the seven indicators analysed, indicating potential goal conflicts between reducing environmental impacts and resource use through reduced beef and milk production while avoiding further loss of biodiversity and ES from agricultural landscapes. However, causal links between ruminants and many of the indicators are still unclear and the distribution of indicator values overlapped to a large extent between the different farm types (see e.g. Fig. 3). Further research is thus needed to identify farms and practices that can promote multiple ES from agricultural landscapes while limiting resource use and negative environmental impacts of agriculture. We did not identify any positive associations between farms specialising in pigs or poultry and our ES indicators, suggesting that such animal production enterprises could be reduced without losing non-provisioning ES from agricultural landscapes. We found positive associations between horses and several indicators. Horse farms have thus far received very limited scientific attention and more research is needed on the contribution of these farms to agricultural landscapes delivering multiple ES.

## Supplementary Information

Below is the link to the electronic supplementary material.

Supplementary file1 (XLSX 62 kb)

Supplementary file2 (PDF 2982kb)

## Footnotes

1 Realisation of ES refers to the process of translating potentials into actual flows of ES that benefit humanity.
