# Supplementary material for: Smaller farm size and ruminant animals are associated with increased supply of non-provisioning ecosystem services
Source: Ambio. 2022 Apr 16;51(9):2025–42. doi: 10.1007/s13280-022-01726-y (PMC9287507; doi:10.1007/s13280-022-01726-y)
Supplement: Supplementary file 2 — Supplementary file2 (PDF 2982kb) [file 13280_2022_1726_MOESM2_ESM.pdf]

Ambio

Supplementary Information
This supplementary information has not been peer reviewed.

Title: **Smaller farm size and ruminant animals are associated with increased supply of non-provisioning ecosystem services**

Authors: Johan O. Karlsson, Pernilla Tidåker, Elin Röös

CONTENTS

Contents 2

Supplementary Figures 3

Detailed description of the ecosystem service indicators 6

LanVar: Landscape variation 6

CrpDst: Cropland distance to non-cropland 6

SSHab: Small-scale habitats 6

Gra: Semi-natural grasslands 7

CrpSeq: Crop sequence 8

RodVar: Roadside variation 10

Acc: Accessibility 10

Visit. Visitors (species observations + social media photos) 10

NatRes. Nature conservation and recreation areas 11

Description of datasets used 12

Integrated Administration and Control System (IACS) database (2013-2019) 12

The Swedish Farm Register (2016) 12

The GSD Property Map in vector format 13

Gridded population data (2019) 13

The Swedish meadow and pasture inventory (TUVA) 13

Artportalen (Swedish Species Observation System; 2013-2019) 13

Flickr photos (2013-2019) 13

Nature reserves, Areas of national interest for recreation and nature conservation and Natura 2000 areas 14

References 14

SUPPLEMENTARY FIGURES

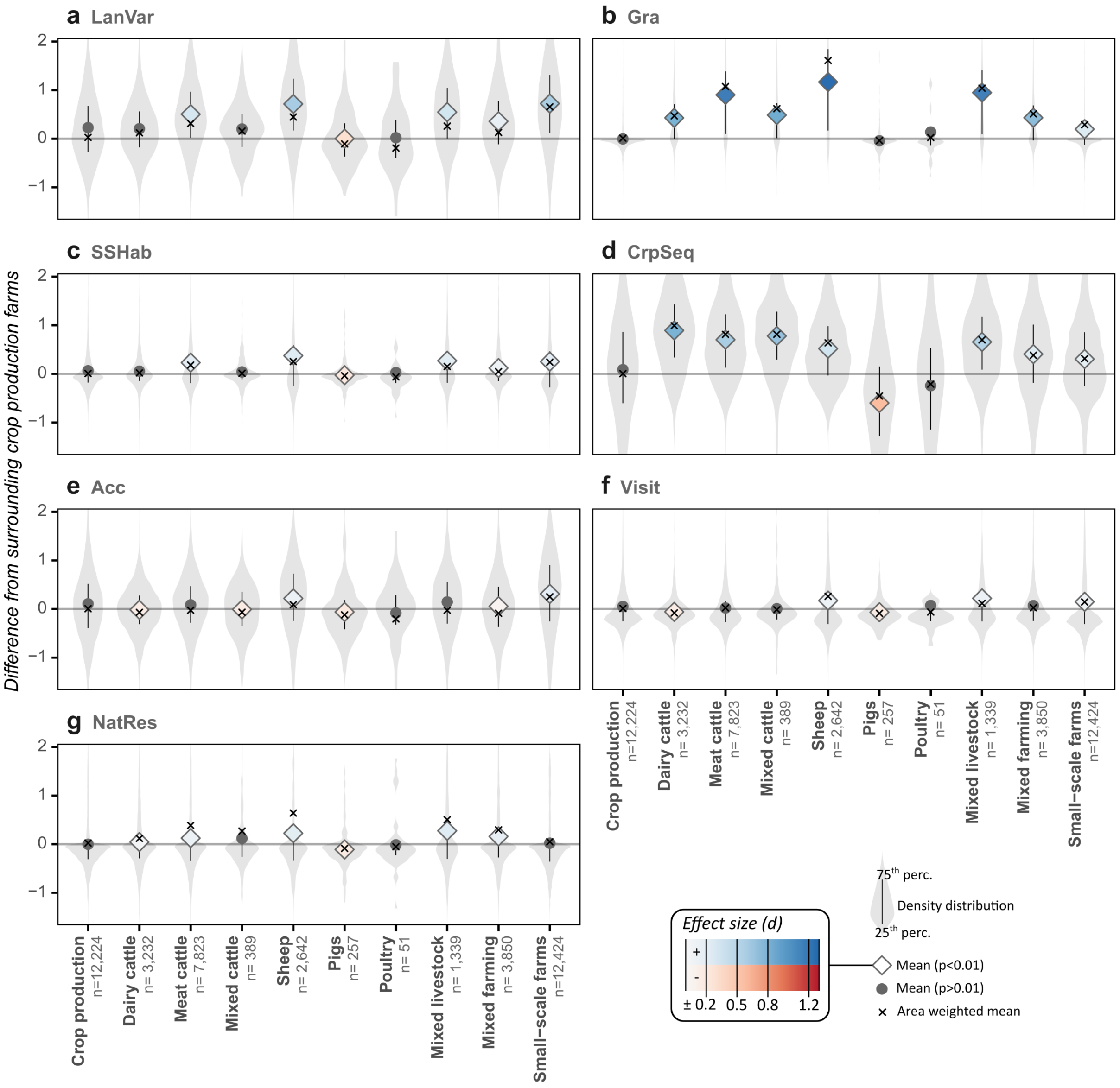

Fig. S1. Effect of farm type on indicator values. The y-axis shows the difference in indicator value from the area-weighted mean indicator value of surrounding crop production farms within a 20-km radius. For each farm type, the interquartile range (error bars) mean (diamonds/circles) and area-weighted mean (crosses) are shown. Diamonds indicate a statistically significant difference in mean difference from the 'Crop production' group (Welch's t-test; p<0.01) and colour indicates effect size (Cohen's d). Note: Mean difference also deviated from zero for the reference farms, due to the area weighting and stochastic effects from how farms are distributed in the landscape.

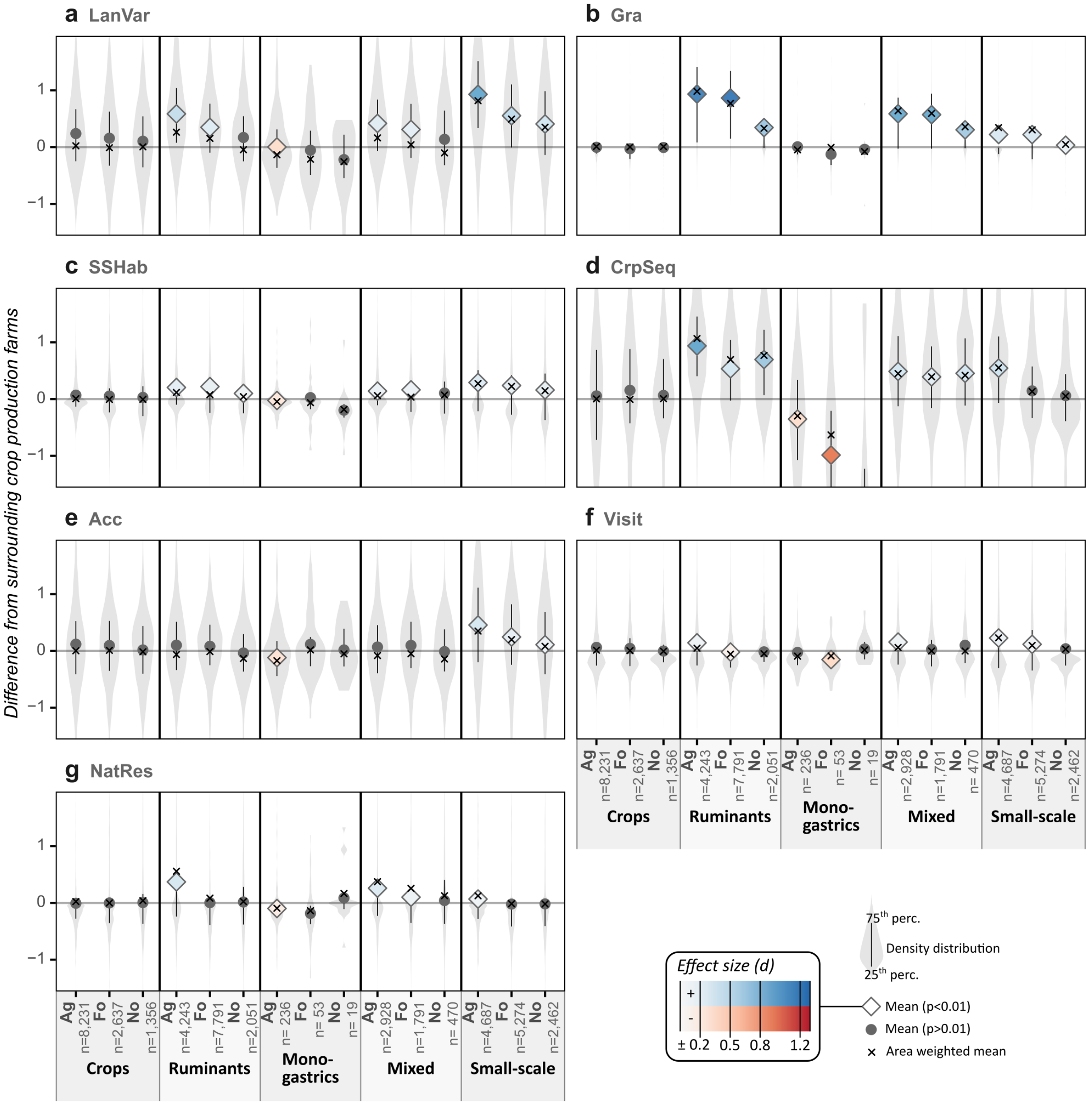

Fig. S2. Effect of farm type on indicator values in the different regions. The y-axis shows the difference in indicator value from the area-weighted mean indicator value of surrounding crop production farms within a 20-km radius. For each farm type, the interquartile range (error bars) mean (diamonds/circles) and area-weighted mean (crosses) are shown. Diamonds indicate a statistically significant difference in mean difference from the 'Crops' group in the same region (Welch's t-test; p<0.01) and colour indicates effect size (Cohen's d). The different regions are represented on the x-axis from left to right; Agricultural regions (Ag), Forested regions (Fo) and Northern Sweden (No). Note: Mean difference also deviated from zero for the reference farms, due to the area weighting and stochastic effects from how farms are distributed in the landscape.

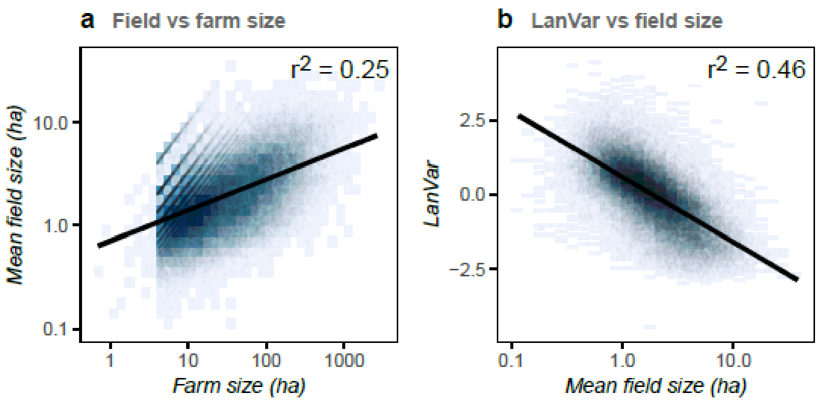

Fig. S3. Correlation between a) mean field size and farm size and b) the landscape variation indicator (LanVar) and mean field size. Pearson least squares regression lines and corresponding r squared values are shown.

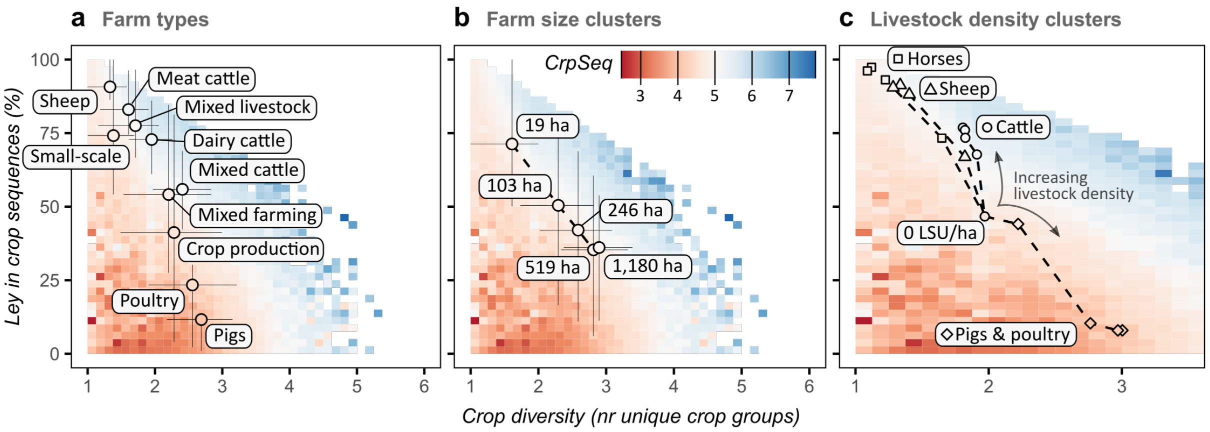

Fig. S4. Observed values on the crop sequence indicator (CrpSeq) depending on the number of unique crop groups (x-axis) and the share of ley (y-axis) in crop sequences. Both crop diversity and the share of ley are calculated as the area-weighted mean across all fields on each farm. For readability, the data are rasterised so that each raster cell represents the mean value on the crop sequence indicator for farms within that cell (indicated with colour). Labelled circles show the mean values for each farm type (panel a), farm size cluster (panel b) and livestock density cluster (panel c). Error bars show the interquartile range of observed values within each group.

DETAILED DESCRIPTION OF THE ECOSYSTEM SERVICE INDICATORS

The following sections provide detailed descriptions of the ecosystem service indicators, including references to literature motivating their relevance as proxies for different ecosystem services and methods for their calculation.

LanVar: Landscape variation

Dataset(s) used: GSD Property Map

Spatial landscape variation, diversity or complexity has relevance for provisioning of several ES, including habitat creation and the presence of pollinating insects (Persson et al. 2015; Rundlöf et al. 2008) and natural enemies of pests (Chaplin-Kramer et al. 2011). Especially forest edges adjacent to open land are important pollinator habitats (Zulian et al. 2013). Although results have been variable and non-linear, the density of edges between contrasting landscape patches (e.g. field margins and forest edges) is generally also seen as beneficial for aesthetic landscape qualities and related ecosystem services (Dronova 2017). Following the method in Andersson et al. (2015), we used the length of edges between different land cover patches represented in the GSD Property Map divided by the farm's total study area as an indicator of landscape variation (**Fig. S5b**).

CrpDst: Cropland distance to non-cropland

Dataset(s) used: GSD Property Map + IACS database

Proximity of cropland to other land uses (e.g. pastures, meadows, forests) can have a positive influence on the presence of pollinators and natural enemies of pests. For example, Bailey et al. (2014) observed that bee abundance and taxa richness in rapeseed fields were both negatively affected by distance to forest edge. Bianchi et al. (2006) notes that "diversity and density of natural enemy populations may decline with increasing distance from non-crop habitats".

We defined this indicator as the average distance from field interiors to the nearest non-crop habitat (other than water or densely built-up areas) within the farm's study area. The indicator was calculated by creating a point grid (10x10m^2) within each cropland parcel and calculating the distance from each point to the nearest non-cropland edge in the GSD property map's land cover layer. The distances were then averaged over the whole farm and multiplied by -1, so that higher values represented better performance on the indicator.

SSHab: Small-scale habitats

Dataset(s) used: IACS database

Small semi-natural habitats within cropland fields (e.g. field islets, clearance cairns and ditches) are important for farmland biodiversity by providing habitats and dispersal corridors (Arlt et al. 2019) and may promote beneficial organisms in much the same way as landscape variation (see LanVar). For example, artificially created grass-sown earth-bank 'islands' in cereal fields have been shown to provide overwintering sites for predatory insects and benefit spring dispersal into the crops (Thomas et al. 1991). For these reasons, e.g. field islets, clearance cairns and small wetlands on agricultural land are currently protected habitats throughout Sweden under environmental law (5 § SFS 1998:1252).

The indicator for small-scale habitats was defined as the number of small-scale habitats within cropland parcels on a farm, divided by the farm's cropland area. We used holes in the agricultural parcel polygons to identify small-scale habitats (**Fig. S5a**). Areas not counting as agricultural, and thus not eligible for agricultural support, are omitted from the parcel polygons (Swedish Board of Agriculture 2010), which leaves holes that are identifiable. The features identifiable in this way include e.g. clearance cairns, field islets/copses, barren rock, shrubs, buildings, permanent areas for storing timber, machinery etc., but generally not linear elements such as ditches, hedgerows or stone walls.

To test the robustness of our method, we manually checked a random selection of identified small-scale habitats (n=115) against aerial photographs using Google Earth and tried to identify the landscape feature represented. The majority of features (n=91; 79%) were identified as field islets or clearance cairns, predominantly with tree or shrub cover but sometimes also with lower vegetation and/or signs of grazing. Ten (9%) features were identified as barren rock, sand or impediments, four (3%) as buildings or constructions surrounded by trees or bushes, two (2%) as ditches and vegetation surrounding ditches, two (2%) as large solitary trees, and one feature was identified as a wetland. Five features (4%) were not possible to distinguish from the surrounding cropland and likely represent impediments or errors in the parcel polygons. The size of identified features in this sample ranged from 5 m^2 to 1.8 ha, with an average of 591 m^2.

Gra: Semi-natural grasslands

Dataset(s) used: IACS database + TUVA

Grasslands, especially semi-natural grasslands, have been linked to a number of ES, including climate regulation, water supply, erosion control, providing habitat for pollinators and natural enemies, as well as non-material services in terms of recreation and cultural heritage (Bengtsson et al. 2019). For example, Öckinger and Smith (2007) showed that richness and density of pollinating insects were higher in field margins closer to semi-natural grasslands in southern Sweden, suggesting that these grasslands are important source habitats, and Alignier et al. (2014) showed that a higher proportion of semi-natural habitats (grasslands, woods and hedges) was positively related to biological pest control. The cultural and recreational values of grasslands and semi-natural grasslands have received less scientific attention and it is often difficult to separate the values of grasslands from the overall landscape structure and "openness" (Bengtsson et al. 2019). However, Marzetti et al. (2011) showed that the majority of visitors at two regional parks in Italy were in favour of preserving semi-natural grasslands as opposed to a rewilding scenario and many were willing to donate for this purpose, with the main motives being the bequest value in conserving the landscape for future generations.

The indicator for semi-natural grasslands was calculated as the area of semi-natural pastures and meadows within the farm's agricultural area (**Fig. S5a**) divided by the total agricultural area. For pastures and meadows represented in the Swedish meadow and pasture inventory (**Fig. S5d**), the area was multiplied by two to account for the higher biological and cultural values of pastures and meadows represented in this database. Areas marked as "needing restauration" or "not relevant" in the database were not included.

CrpSeq: Crop sequence

Dataset(s) used: IACS database

Diverse and well-designed crop sequences, especially those including perennial forage crops (leys), can provide ES by building soil organic matter and fertility (Albizua et al. 2015; Tiemann et al. 2015), preventing diseases, pests and weeds (Ball et al. 2005; Rusch et al. 2013) and supplying nitrogen (Nevens & Reheul 2002), thus reducing the need for external inputs such as chemical plant protection and fertilisers to achieve a certain yield. The crop sequence indicator was calculated using the methodology presented by Leteinturier et al. (2006), but adapted to Swedish conditions and generalised to broad crop groups instead of individual crops. The indicator is calculated as the product of three variables that reflect the beneficial or detrimental effect of the previous crop on the next one in the sequence (k_p), the difference between observed and recommended return times of re-occurring crops in the sequence (k_r), and the diversity of crops in the sequence (k_d). The k_p value is the main component of the indicator and ranges from 1 (worst) to 6 (best), while the k_r (range 0.2-1.2) and k_d (range 1.0-1.39) are factors adjusting this score up or down. For a more detailed description of the methodology and calculations, see Leteinturier et al. (2006). The indicator was calculated for each individual cropland parcel on a farm and then summed to an area-weighted mean for the entire farm as:

$$CrpSeq = \frac{\sum A \times k_{p\,mean} \times k_{r\,mean} \times k_d}{\sum A} \quad \text{(S1)}$$

where A is the area of each individual parcel on a farm, $k_{p\,mean}$ is the average preceding crop value for the crop sequence, $k_{r\,mean}$ is the mean return time coefficient and k_d is the crop diversity coefficient.

Inclusion of ley cultivation and fallow periods in crop sequences generally has positive effects on the crop sequence indicator. However, as many ES associated with ley or fallow periods (e.g. providing nitrogen and pest and weed suppression) are only realised if they are integrated with arable crops in a sequence, we introduced a cut-off at year three of consecutive ley/fallow, after which the k_p value was reduced (shown in brackets in **Table S2**). In this way, crop sequences that integrate ley and fallow with other crops were favoured over sequences with long ley or fallow periods.

Crop sequences were derived for a seven-year period (2013-2019) by geographical overlay of each farm's cropland parcels in 2016 with parcel data for the preceding and following years. This allowed us to track cropland parcels through time, but disregard whether a cropland parcel was farmed by the same farmer throughout the whole period or not. The indicator was only calculated for farms where it was possible to derive complete crop sequences for at least 50% of the farm's cropland area. **Tables S1 and S2** show the crop groups and corresponding parameter values used. For illustrative purposes, **Table S3** provides results from a selected number of observed sequences.

Table S1. Crop types used calculating the crop sequence indicator

Crop type	Example of crops included	Crop codes
Winter cereals (WC)	Winter wheat, rye, triticale, winter barley	1, 4, 7, 8
Spring cereals (SC)	Spring barley, spring wheat, oats, maize	2, 3, 5, 9, 11, 12, 13, 14, 15, 16, 29
Pulses (Pu)	Peas, beans	30, 31, 32, 33, 34, 35, 37, 38, 39, 43
Brassicaceae (Br)	Spring/winter rapeseed, mustard, oil radish	20, 21, 22, 23, 25, 26, 27, 28
Ley (Le)	Different mixtures of grasses and legumes	6, 36, 49, 50, 57, 58, 59, 62, 63, 80, 81
Beets (Be)	Sugar beet, fodder beet	47, 48
Potatoes (Po)	-	45, 46
Flax (Fl)	-	40, 41
Woody crops (Wo)	Short rotation coppice	65, 67, 68, 71, 72, 78, 83
Other (Ot)	Horticultural crops	10, 24, 42, 70, 74, 79, 85, 86, 87, 88
Fallow (Fa)	-	60, 69

Table S2. Values used for the parameters k_p and t_R. Numbers in brackets refer to k_p values used for year 4 and onwards of consecutive ley or fallow. See Table S1 for abbreviations

Crop	**Preceding crop value (k_p)**											**Minimum return time (t_R)**
	WC	SC	Pu	Br	Le	Be	Po	Fl	Wo	Ot	Fa	
WC	1	4	6	5	4	5	5	5	4	5	2	2
SC	3	2	4	3	5	5	4	4	4	4	2	2
Pu	5	5	1	1	6	4	5	3	5	5	3	6
Br	5	5	5	1	5	4	1	4	5	1	4	6
Le	6	6	6	6	6(4)	6	6	6	6	6	6	1
Be	4	4	6	2	3	1	5	4	3	5	3	3
Po	5	5	3	3	2	3	1	3	2	1	2	5
Fl	5	5	1	2	1	1	1	1	1	1	2	6
Wo	6	6	6	6	6	6	6	6	6	6	6	1
Ot	5	5	3	3	2	3	1	3	2	1	2	5
Fa	4	4	4	3	5	6	6	6	5	6	5(3)	1

Table S3. A selected number of observed crop sequences and their resulting CrpSeq-value. See Table S1 for abbreviations

Crop sequence 2013	2014	2015	2016	2017	2018	2019	$k_{p\ mean}$	$k_{r\ mean}$	k_d	**CrpSeq**
Be	SC	WC	Le	Br	WC	Le	5.17	1.20	1.30	**8.06**
Be	SC	Le	Br	WC	Le	Le	5.50	1.10	1.30	**7.87**
Fa	Le	WC	Le	Le	SC	Le	5.50	1.13	1.25	**7.79**
SC	WC	Be	SC	Br	WC	Be	4.50	1.20	1.25	**6.75**
Le	Fa	Fa	SC	Le	Le	Le	5.00	1.05	1.20	**6.30**
Le	Le	Le	Le	SC	Le	Le	5.50	1.04	1.10	**6.29**
Le	Le	Le	Le	Le	Le	Le	4.67	1.00	1.00	**4.67**
Le	WC	SC	SC	SC	Le	Le	3.83	0.95	1.20	**4.37**
Fa	Fa	Fa	Fa	Fa	Fa	SC	3.50	1.03	1.10	**3.98**
SC	SC	SC	SC	Le	Le	Le	4.00	0.88	1.10	**3.87**
WC	WC	SC	WC	SC	SC	SC	2.50	0.88	1.10	**2.42**
SC	SC	WC	SC	SC	SC	SC	2.50	0.84	1.10	**2.31**
SC	SC	SC	SC	SC	SC	SC	2.00	0.80	1.00	**1.60**

RodVar: Roadside variation

Dataset(s) used: GSD Property Map

Although results have been variable, measures of landscape diversity generally show positive impacts on perceived landscape beauty and visual quality (Dramstad et al. 2006; Dronova 2017) and roads and paths are the primary means by which people move through a landscape. The indicator was calculated as the number of land cover patches intersected by or adjacent (within 25 m) to roads and paths (excluding motorways and railways), divided by the total length of roads within the study area, based on the method presented by Andersson et al. (2015).

Acc: Accessibility

Dataset(s) used: GSD Property Map + Gridded population data

Accessibility is an important precondition for ES related to recreation and tourism and population density has previously been used in indicators for recreational ES. For example Qiu and Turner (2013) factored the population within a 10-km radius into their indicator for forest recreation. We based our indicator on Andersson et al. (2015), who used the fraction of a landscape within 100 m from roads (**Fig. S5c**) as an indicator for accessibility. To account not only for physical infrastructure, but also for the closeness of farmland to people, we factored in the population density around each farm as:

$$Acc = (\frac{A_{acc}}{A_{sa}})_{scaled} + (Pop)_{scaled} \tag{S2}$$

where A_{acc} is the area within 100 m from roads, A_{sa} is the total study area, Pop is the population density within a 10-km buffer around the farm's study area and the 'scaled' subscript indicates that both terms were scaled to zero mean and one standard deviation prior to addition. The scaling was performed in order to give both terms equal weight in the final indicator value.

Visit: Visitors (species observations + social media photos)

Dataset(s) used: Artportalen + Flickr photos

Georeferenced social media uploads are increasingly used to quantify recreational use of nature areas and their cultural ecosystem services (Le Clec'h et al. 2019; Queiroz et al. 2015; Raudsepp-Hearne et al. 2010; Turner et al. 2014). We used species observations reported to *Artportalen* and photo uploads to *Flickr* and counted the number of unique users uploading photos or reporting species observations within each farm's study area during the period 2013-2019 divided by the total study area (**Fig. S5c**). In order to give the two data sources equal weight in the final indicator, we calculated the number of *Artportalen* and *Flickr* users per hectare separately and scaled the values to zero mean and one standard deviation prior to summation. We excluded bird observations from the *Artportalen* data, to avoid biases due to migratory bird paths and resting locations that are not directly influenced by the landscape around a specific farm.

NatRes: Nature conservation and recreation areas

Dataset(s) used: Nature reserves, Areas of national interest for recreation and nature conservation, Natura 2000 areas

We calculated this indicator as the total area of nature reserves, areas protected under the EU Habitats and Birds Directives (Natura 2000), and areas classified as of national interest for recreation and nature conservation within each farm's study area divided by the total study area (**Fig. S5d**). In cases where an area was protected or classified in more than one way, that area was counted once for each classification. For example, if a farm had a 40-ha study area with 20 ha designated as a nature reserve, 10 ha protected under Natura 2000 and 20 ha classified as of national interest for recreation, it received a value of (20 + 10 + 20) / 40 = 1.25 for this indicator, even though these areas partly or completely overlap.

This indicator rests on the assumption that areas protected or classified according to the data sources used correlate with higher levels of biological and recreational values than the surrounding landscape and thus supply more ES related to cultural and recreational values and habitat creation. Protected and otherwise classified areas have previously been used as proxies for different ES, e.g. Queiroz et al. (2015) used areas classified as being of national interest for recreation as an indicator for ES related to nature recreation and Turner et al. (2014) used areas protected under the Danish Nature Conservation Act as a proxy for "sense of place".

DESCRIPTION OF DATASETS USED

The following sections provide descriptions of the datasets used to calculate the different ecosystem service indicators. **Fig. S5** provides an illustrative example of the datasets used for a selected farm.

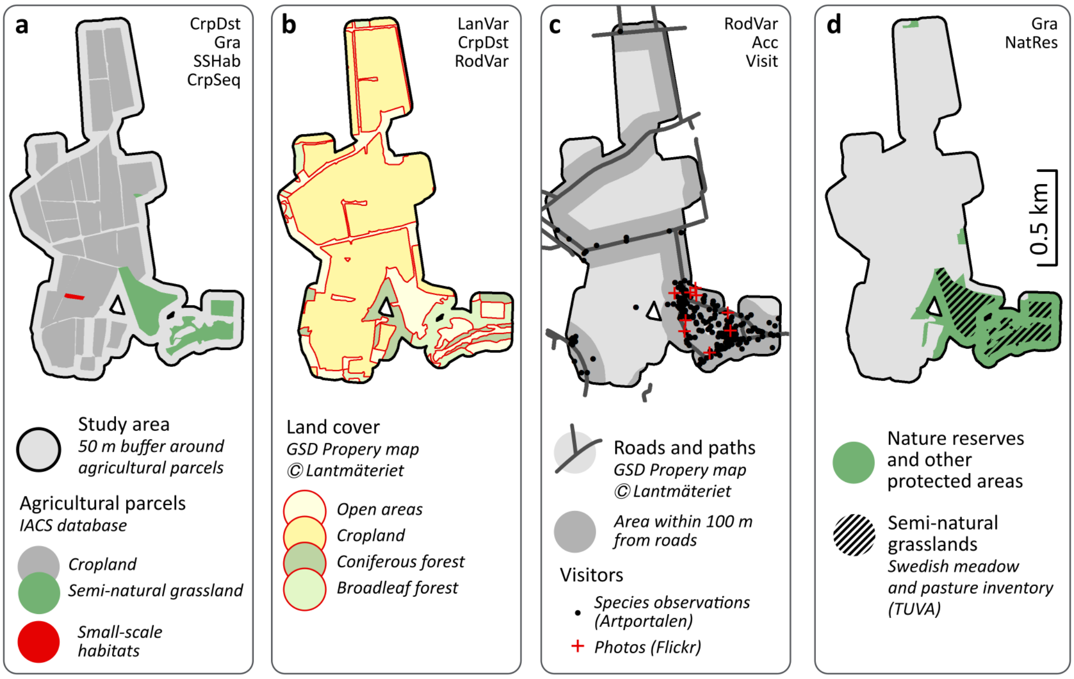

Fig. S5. Illustration of a farm's study area and map layers used to calculate the indicators. Panel (a) shows the farm's cropland (dark grey) and semi-natural grassland (green) parcels according to the IACS database, the farm's study area (light grey with black outline) and identified small-scale habitats in cropland (red). Panel (b) shows the land cover map with borders around land cover patches highlighted in red. Panel (c) shows roads and paths (grey lines) and the area of the farm's study area within 100 m from roads and paths (dark grey). This panel also shows species observations to *Artportalen* (black dots) and photo uploads to *Flickr* (red crosses) within the farm's study area. Panel (d) shows the areas of the farm's study area that fall within a nature reserve or other protected area (green) as well as the area of semi-natural grasslands that are represented in the Swedish meadow and pasture inventory (TUVA; diagonal hatch). In the top right of each panel indicators relying on the data layers shown are indicated. The specific farm depicted here was selected as all data layers were represented (e.g. many farms did not have any visitor points or protected areas) and it was of a suitable size for plotting.

Integrated Administration and Control System (IACS) database (2013-2019)

Data provider: Swedish Board of Agriculture

The IACS database contains data on all agricultural parcels in Sweden for which farmers have applied for agricultural support. Each parcel is represented by a polygon with information on crop grown each year and a farmer identification number.

The Swedish Farm Register (2016)

Data provider: Swedish Board of Agriculture

This is a register of Swedish farms used for the preparation of national agricultural statistics. In the Swedish Farm Register, a farm is included if it:

- farms at least 2 ha cropland or
- at least 5 ha agricultural land or

- keeps a minimum number of animals (10 cattle, 10 sows, 50 pigs, 20 sheep or 1,000 poultry) or
- commercially produces horticultural crops on at least 2,500 m^2 or 200 m^2 greenhouse.

For each farm in the farm register, information is available on e.g. area farmed, animals on the farm, farm typology class and farmer identification number(s). Since farmers may decide to split their operations over different economic entities, there are frequently several farmer identification numbers tied to a single farm. Through the farmer identification numbers, each farm in the farm register can be tied to land parcels in the IACS database.

The GSD Property Map in vector format

Data provider: Lantmäteriet (the Swedish mapping, cadastral and land registration authority)

The GSD Property Map is a detailed map of Sweden including e.g. land cover types and road networks. Land cover in the property map is categorised as arable, open (including pastures and meadows), forest (coniferous, broadleaf or mixed), water, built-up area etc. The road layer includes roads, as well as bicycle tracks and larger paths/walking trails.

Gridded population data (2019)

Data provider: SCB (Statistics Sweden)

This dataset contains data on the number of inhabitants per 1x1 km^2 grid cell based on the Swedish register of the total population.

The Swedish meadow and pasture inventory (TUVA)

Data provider: Swedish Board of Agriculture

This database contains data on a large number of Swedish semi-natural grasslands. Each grassland object is represented by a polygon and contains information on e.g. cultural and biological values and its land use classification (pasture, meadow, needing restoration or not relevant).

Artportalen (Swedish Species Observation System; 2013-2019)

Data provider: SLU Artdatabanken (SLU Swedish Species Information Centre)

This is an open database where anyone can report species observations. For each observation, data on e.g. observer, geographical location, observation date and taxon are available.

Flickr photos (2013-2019)

Data provider: Flickr

Flickr is an online image hosting service and community. Through an application programming interface (API), all publicly visible and geotagged photos can be queried based on geographical location and information on e.g. owner, date, coordinates, photo title, tags etc. can be retrieved.

Nature reserves, Areas of national interest for recreation and nature conservation and Natura 2000 areas

Data provider: Swedish Environmental Protection Agency

These datasets include polygons that represent 1) nature reserves protected under Swedish environmental law, 2) areas of national interest for recreation, which are areas with distinct natural or cultural qualities and good accessibility for a large number of visitors, 3) areas of national interest for nature conservation, which are areas that intend to represent the main characteristics and diversity of Swedish nature, and 4) Natura 2000 areas protected under the EU Habitats (92/43/EEG) or Birds Directive (79/409/EEG).

REFERENCES

Albizua, A., Williams, A., Hedlund, K., & Pascual, U. (2015). Crop rotations including ley and manure can promote ecosystem services in conventional farming systems. *Applied Soil Ecology, 95*, 54-61. doi:https://doi.org/10.1016/j.apsoil.2015.06.003

Alignier, A., Raymond, L., Deconchat, M., Menozzi, P., Monteil, C., Sarthou, J.-P., . . . Ouin, A. (2014). The effect of semi-natural habitats on aphids and their natural enemies across spatial and temporal scales. *Biological Control, 77*, 76-82. doi:https://doi.org/10.1016/j.biocontrol.2014.06.006

Andersson, E., Nykvist, B., Malinga, R., Jaramillo, F., & Lindborg, R. (2015). A social–ecological analysis of ecosystem services in two different farming systems. *AMBIO, 44*(1), 102-112. doi:https://doi.org/10.1007/s13280-014-0603-y

Arlt, D., Josefsson, J., Kindström, M., & Glimskär, A. (2019). *Indikatorer för småbiotoper i odlingslandskapet [Indicators for small-scale habitats in the agricultural landscape]*. Retrieved from https://core.ac.uk/download/pdf/287728664.pdf

Bailey, S., Requier, F., Nusillard, B., Roberts, S. P. M., Potts, S. G., & Bouget, C. (2014). Distance from forest edge affects bee pollinators in oilseed rape fields. *Ecology and Evolution, 4*(4), 370-380. doi:https://doi.org/10.1002/ece3.924

Ball, B. C., Bingham, I., Rees, R. M., Watson, C. A., & Litterick, A. (2005). The role of crop rotations in determining soil structure and crop growth conditions. *Canadian Journal of Soil Science, 85*(5), 557-577. doi:https://doi.org/10.4141/S04-078

Bengtsson, J., Bullock, J. M., Egoh, B., Everson, C., Everson, T., O'Connor, T., . . . Lindborg, R. (2019). Grasslands—more important for ecosystem services than you might think. *Ecosphere, 10*(2), e02582. doi:https://doi.org/10.1002/ecs2.2582

Bianchi, F. J. J. A., Booij, C. J. H., & Tscharntke, T. (2006). Sustainable pest regulation in agricultural landscapes: a review on landscape composition, biodiversity and natural pest control. *Proceedings of the Royal Society B: Biological Sciences, 273*(1595), 1715-1727. doi:https://doi.org/10.1098/rspb.2006.3530

Chaplin-Kramer, R., O'Rourke, M. E., Blitzer, E. J., & Kremen, C. (2011). A meta-analysis of crop pest and natural enemy response to landscape complexity. *Ecology Letters, 14*(9), 922-932. doi:https://doi.org/10.1111/j.1461-0248.2011.01642.x

Dramstad, W. E., Tveit, M. S., Fjellstad, W. J., & Fry, G. L. A. (2006). Relationships between visual landscape preferences and map-based indicators of landscape structure. *Landscape and Urban Planning, 78*(4), 465-474. doi:https://doi.org/10.1016/j.landurbplan.2005.12.006

Dronova, I. (2017). Environmental heterogeneity as a bridge between ecosystem service and visual quality objectives in management, planning and design. *Landscape and Urban Planning, 163*, 90-106. doi:https://doi.org/10.1016/j.landurbplan.2017.03.005

Le Clec'h, S., Finger, R., Buchmann, N., Gosal, A. S., Hörtnagl, L., Huguenin-Elie, O., . . . Huber, R. (2019). Assessment of spatial variability of multiple ecosystem services in grasslands of

different intensities. *Journal of Environmental Management, 251*, 109372. doi:https://doi.org/10.1016/j.jenvman.2019.109372

Leteinturier, B., Herman, J. L., Longueville, F. d., Quintin, L., & Oger, R. (2006). Adaptation of a crop sequence indicator based on a land parcel management system. *Agriculture, Ecosystems & Environment, 112*(4), 324-334. doi:https://doi.org/10.1016/j.agee.2005.07.011

Marzetti, S., Disegna, M., Villani, G., & Speranza, M. (2011). Conservation and recreational values from semi-natural grasslands for visitors to two Italian parks. *Journal of Environmental Planning and Management, 54*(2), 169-191. doi:https://doi.org/10.1080/09640568.2010.505792

Nevens, F., & Reheul, D. (2002). The nitrogen- and non-nitrogen-contribution effect of ploughed grass leys on the following arable forage crops: determination and optimum use. *European Journal of Agronomy, 16*(1), 57-74. doi:https://doi.org/10.1016/S1161-0301(01)00115-0

Persson, A. S., Rundlöf, M., Clough, Y., & Smith, H. G. (2015). Bumble bees show trait-dependent vulnerability to landscape simplification. *Biodiversity and Conservation, 24*(14), 3469-3489. doi:https://doi.org/10.1007/s10531-015-1008-3

Qiu, J., & Turner, M. G. (2013). Spatial interactions among ecosystem services in an urbanizing agricultural watershed. *Proceedings of the National Academy of Sciences, 110*(29), 12149-12154. doi:https://doi.org/10.1073/pnas.1310539110

Queiroz, C., Meacham, M., Richter, K., Norström, A. V., Andersson, E., Norberg, J., & Peterson, G. (2015). Mapping bundles of ecosystem services reveals distinct types of multifunctionality within a Swedish landscape. *AMBIO, 44*(1), 89-101. doi:https://doi.org/10.1007/s13280-014-0601-0

Raudsepp-Hearne, C., Peterson, G. D., & Bennett, E. M. (2010). Ecosystem service bundles for analyzing tradeoffs in diverse landscapes. *Proceedings of the National Academy of Sciences, 107*(11), 5242-5247. doi:https://doi.org/10.1073/pnas.0907284107

Rundlöf, M., Nilsson, H., & Smith, H. G. (2008). Interacting effects of farming practice and landscape context on bumble bees. *Biological Conservation, 141*(2), 417-426. doi:https://doi.org/10.1016/j.biocon.2007.10.011

Rusch, A., Bommarco, R., Jonsson, M., Smith, H. G., & Ekbom, B. (2013). Flow and stability of natural pest control services depend on complexity and crop rotation at the landscape scale. *Journal of Applied Ecology, 50*(2), 345-354. doi:https://doi.org/10.1111/1365-2664.12055

Swedish Board of Agriculture. (2010). *Instruktion för fältinventering av brukarblock, Version 5 [Instruction for field inventory of agricultural parcels, Version 5]*. Retrieved from https://djur.jordbruksverket.se/download/18.32b12c7f12940112a7c80009596/1370040765271/OVR159-5_Instruktion_v5,%20beslutad.pdf

Thomas, M. B., Wratten, S. D., & Sotherton, N. W. (1991). Creation of 'Island' Habitats in Farmland to Manipulate Populations of Beneficial Arthropods: Predator Densities and Emigration. *Journal of Applied Ecology, 28*(3), 906-917. doi:https://doi.org/10.2307/2404216

Tiemann, L. K., Grandy, A. S., Atkinson, E. E., Marin-Spiotta, E., & McDaniel, M. D. (2015). Crop rotational diversity enhances belowground communities and functions in an agroecosystem. *Ecology Letters, 18*(8), 761-771. doi:https://doi.org/10.1111/ele.12453

Turner, K. G., Odgaard, M. V., Bocher, P. K., Dalgaard, T., & Svenning, J. C. (2014). Bundling ecosystem services in Denmark: Trade-offs and synergies in a cultural landscape. *Landscape and Urban Planning, 125*, 89-104. doi:https://doi.org/10.1016/j.landurbplan.2014.02.007

Zulian, G., Maes, J., & Paracchini, M. L. (2013). Linking Land Cover Data and Crop Yields for Mapping and Assessment of Pollination Services in Europe. *Land, 2*(3), 472-492.

Öckinger, E., & Smith, H. G. (2007). Semi-natural grasslands as population sources for pollinating insects in agricultural landscapes. *Journal of Applied Ecology, 44*(1), 50-59. doi:https://doi.org/10.1111/j.1365-2664.2006.01250.x
